# Ubiquitin and its relatives as wizards of the endolysosomal system

**DOI:** 10.1242/jcs.260101

**Published:** 2023-02-24

**Authors:** Ilana Berlin, Aysegul Sapmaz, Virginie Stévenin, Jacques Neefjes

**Affiliations:** Oncode Institute, Department of Cell and Chemical Biology, Leiden University Medical Center LUMC, Einthovenweg 20, 2300RC Leiden, The Netherlands

**Keywords:** Bacterial infection, Endosomes, Membrane contact sites, Membrane dynamics, Ubiquitin

## Abstract

The endolysosomal system comprises a dynamic constellation of vesicles working together to sense and interpret environmental cues and facilitate homeostasis. Integrating extracellular information with the internal affairs of the cell requires endosomes and lysosomes to be proficient in decision-making: fusion or fission; recycling or degradation; fast transport or contacts with other organelles. To effectively discriminate between these options, the endolysosomal system employs complex regulatory strategies that crucially rely on reversible post-translational modifications (PTMs) with ubiquitin (Ub) and ubiquitin-like (Ubl) proteins. The cycle of conjugation, recognition and removal of different Ub- and Ubl-modified states informs cellular protein stability and behavior at spatial and temporal resolution and is thus well suited to finetune macromolecular complex assembly and function on endolysosomal membranes. Here, we discuss how ubiquitylation (also known as ubiquitination) and its biochemical relatives orchestrate endocytic traffic and designate cargo fate, influence membrane identity transitions and support formation of membrane contact sites (MCSs). Finally, we explore the opportunistic hijacking of Ub and Ubl modification cascades by intracellular bacteria that remodel host trafficking pathways to invade and prosper inside cells.

## Teaser

**Summary:** A Review of ubiquitylation and its biochemical relatives as regulators of the endolysosomal system and its hijacking by intracellular bacteria in order for them to prosper inside cells.

## Introduction

Compartmentalization of cellular processes into membrane-enclosed organelles defines the eukaryotic life form and underlies the emergence of complex (multi)cellular systems. In a living cell, compartmentalization is necessarily dynamic and thus exhibits not only spatial but also temporal constraints. This notion is exemplified by the endolysosomal system – an interactive network of diverse vesicular carriers whose identity, contents and location are continuously subject to change. To make sense of this complexity, reversible post-translational modifications (PTMs) with ubiquitin (Ub) and ubiquitin-like (Ubl) proteins come aptly into play.

**Fig. 1.**
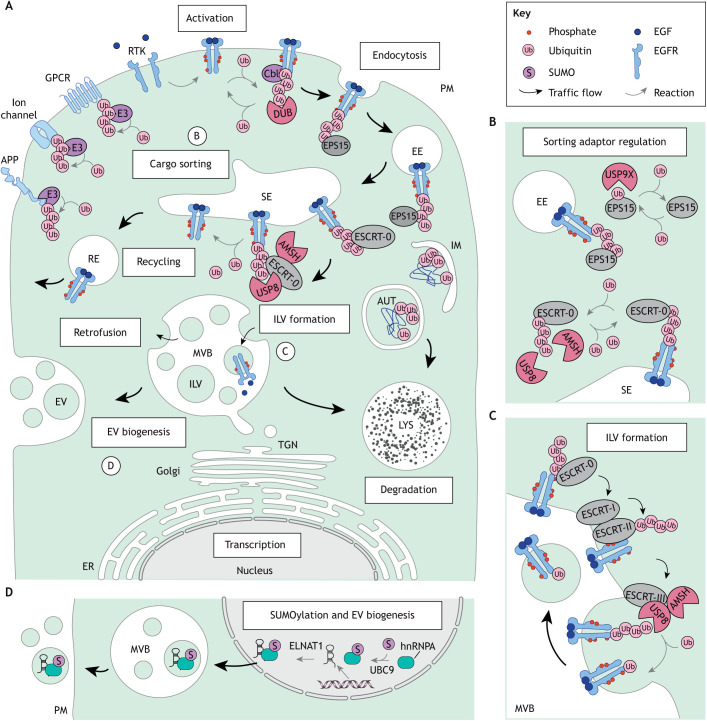
**Ubiquitylation drives lysosomal turnover of the cell surface proteome.** (A) Ubiquitin (Ub) modifications direct cell surface proteins for degradation, as exemplified by the epidermal growth factor receptor (EGFR) whose ligand-induced activation sets in motion its journey to lysosomes. Cargoes endocytosed in early endosomes (EEs) are partitioned between the degradative and recycling membrane pathways at the sorting endosome (SE). Ligand binding triggers EGFR phosphorylation and recruitment of the E3 ubiquitin ligase Cbl for receptor ubiquitylation. Whereas receptors lacking Ub marks traffic back to the plasma membrane (PM) via recycling endosomes (REs), ubiquitylated receptors are recognized by endosomal adaptors and incorporated into intralumenal vesicles (ILVs) of multivesicular bodies (MVBs). Subsequent fusion of MVBs with proteolytic lysosomes (LYS) commits ILV cargoes for degradation. The latter fate can, however, be averted by timely action of DUBs at various steps along the endosomal trafficking route. Besides cargo degradation, other ILV pathways exist, including return to the limiting membrane (LM) via retrofusion and release as extracellular vesicles (EVs). The autophagy pathway also feeds into the endolysosomal system via double-membrane autophagosomes (AUT) that form upon sealing of the isolation membrane (IM) around ubiquitylated cytosolic cargoes. Processes labeled B–D are shown in more detail in the other panels. (B) Ub-dependent functions of endosomal sorting adaptors are regulated by reversible ubiquitylation. Monoubiquitylation of EPS15 inhibits recognition of ubiquitylated cargoes, and deubiquitylation by USP9x restores this function. ESCRT-0 function is also positively regulated by its associated DUBs USP8 and AMSH. (C) Sequestration of ubiquitylated cargoes on ILVs is orchestrated by sequential actions of the ESCRT-0, -I, -II, and -III complexes. ESCRT-associated deubiquitylation during ILV formation ensures Ub homeostasis on endosomal membranes and maintains the cellular Ub pool. (D) SUMOylation of the RNA-binding protein hnRNPA facilitates incorporation of the long noncoding RNA ELNAT1 into ILVs for release in EVs.

Following internalization from the plasma membrane (PM), early endosomes (EEs) embark on a journey of progressive maturation (Bakker et al., 2017) characterized by luminal acidification and lipid bilayer remodeling (Maxfield and Yamashiro, 1987; Wang et al., 2019) (Fig. 1A). Maturing or sorting endosomes (SEs) are complex organelles (Klumperman and Raposo, 2014; Scott et al., 2014) that move swiftly through cellular space and sort membranes to a variety of destinations (Naslavsky and Caplan, 2018). Tubules emanating from these compartments accumulate cargoes intended for recycling back to the PM and other organelles (Cullen and Steinberg, 2018). Concomitantly, cargoes destined for lysosomal degradation or secretion become sequestered on intralumenal vesicles (ILVs) during multivesicular body (MVB) biogenesis (Gruenberg, 2020). Fusion of MVBs, also referred to as late endosomes (LEs), with proteolytic lysosomes commits ILV cargoes for degradation, whereas fusion of the same compartments with the PM expels ILVs as extracellular vesicles (EVs) (Gurung et al., 2021). A fraction of ILVs, however, manages to avoid the terminal fates of degradation and secretion by fusing back to the limiting membrane of the MVB through a process termed retrofusion (Perrin et al., 2021). MVBs and lysosomes also closely cooperate with the macroautophagy pathway to mediate clearance of cytoplasmic materials not suitable for degradation by the proteasome, which include large protein aggregates, damaged organelles and even invading pathogens (Birgisdottir and Johansen, 2020). Targeting cytosolic cargoes to the lysosome requires their sequestration into double-membrane vesicles termed autophagosomes, which subsequently fuse with proteolytic endosomes or lysosomes to achieve cargo clearance and nutrient recycling (Nakamura and Yoshimori, 2017). Together with the autophagy pathway, the endolysosomal system oversees signal transduction (Di Fiore and von Zastrow, 2014), nutrient sensing and metabolism (Efeyan et al., 2015; Lim and Zoncu, 2016), maintenance of cell polarity (Shafaq-Zadah et al., 2020), mitotic progression (Carlton et al., 2020), intercellular communication (Maas et al., 2017) and other processes. Given that cellular physiology is intimately connected to organismal wellbeing, aberrant endocytic traffic results in a broad spectrum of pathologies ranging from cancer (Mellman and Yarden, 2013) to neurodegeneration (Fleming et al., 2022; Lie and Nixon, 2019). Besides its homeostatic functions, the endolysosomal system is also the site of entry for pathogenic bacteria and viruses into the host cell (Cossart and Helenius, 2014). Consequently, endocytic compartments are ideally positioned to facilitate antigen processing and presentation (Roche and Furuta, 2015), and disturbances in endosome function are strongly linked to immune deficiencies (Unanue et al., 2016).

Healthy traffic flow along the endolysosomal tract relies heavily on PTMs with Ub and its family members, which help designate cargo fate and direct actions of sorting and trafficking machineries in a spatially and temporally resolved manner. Ub constitutes a uniquely versatile protein modifier whose attachment to substrates is mediated by a multi-step reaction cascade (Pickart, 2001; Varshavsky, 2012) involving sequential engagement of activating (E1), conjugating (E2) (Stewart et al., 2016) and ligating (E3) enzymes (Zheng and Shabek, 2017). The two largest families of Ub ligases, the homologous to E6-AP C-terminus (HECT) and really interesting new gene (RING), differ in their mode of Ub transfer (Metzger et al., 2012; Toma-Fukai and Shimizu, 2021), but give rise to indistinguishable reaction products – Ub moieties covalently attached to (typically) lysine residues of select substrates. Because many proteins feature more than one solvent exposed lysine, the choice of modification site is often determinant of downstream outcomes. Ub itself possesses seven lysine residues (K6, K11, K27, K29, K33, K48 and K63) and an N-terminal methionine, all of which can serve as Ub acceptor sites and thus support polyubiquitin linkages of varying lengths and shapes (Akutsu et al., 2016). This spectacular diversity of Ub signals (Yau and Rape, 2016) can exert a wide range of consequences that include marking proteins for degradation (Ciechanover, 2015) or altering their interaction landscapes and activities in a matter of minutes (Swatek and Komander, 2016). The information encoded by various types of ubiquitylation can in turn be altered or removed by deubiquitylating enzymes (DUBs), hence determining how long a given Ub modification persists. Human DUBs, of which Ub-specific proteases (USPs) comprise the largest and most promiscuous family (Clague et al., 2019), are outnumbered by ligases by nearly 10-fold (Sahtoe and Sixma, 2015). Consequently, many DUBs engage in a variety of partnerships and influence diverse cellular pathways, including endocytosis and membrane trafficking (Mevissen and Komander, 2017). Ubl cascades follow the paradigm of ubiquitylation but utilize Ubl-specific activating, conjugating and ligating enzymes, and proteases (Cappadocia and Lima, 2018). Prominent among the Ubls implicated in endocytosis are small Ub-like modifier (SUMO) proteins (Su et al., 2020) and interferon-stimulated gene 15 (ISG15) (Perng and Lenschow, 2018). In this Review, we cover recent advancements in our understanding of how modifications with Ub and Ubls inform the organization, behavior and function of the endolysosomal system and delve into their exploitation by intracellular bacterial pathogens seeking to establish and maintain a fertile reproductive niche.

## Ub and Ubls in endocytosis and cargo sorting

### Ubiquitylation of the cell surface proteome

The first discoveries implicating ubiquitylation as a regulatory signal in endocytosis came from studies in yeast demonstrating that conjugation of a single Ub moiety to cell surface receptors is sufficient to trigger their internalization and direct them to the vacuole for degradation (Hicke and Riezman, 1996; Shih et al., 2000). Contemporaneously, connections between ubiquitylation and endocytosis were reported in mammalian cells (Levkowitz et al., 1996; Levkowitz et al., 1998). In the decades that followed, general mechanisms underlying Ub-dependent commitment of PM proteins for proteolysis across the eukaryotic kingdom began to emerge (Acconcia et al., 2009; Weinberg and Drubin, 2014). In yeast, the HECT E3 Rsp5 is the predominant Ub ligase responsible for quality control of PM proteins (Sardana et al., 2019). Rsp5 is an essential gene product that achieves substrate specificity by partnering with an array of arrestin-related trafficking adaptors, or ARTs (Barata-Antunes et al., 2021; Guiney et al., 2016), which enable controlled recognition and ubiquitylation of diverse cell surface receptors, transporters and channels (Savocco et al., 2019; Sen et al., 2020; Wawrzycka et al., 2019). In mammalian cells, ligand-induced ubiquitylation of receptor tyrosine kinases (RTKs) (Fig. 1A–C) and G protein-coupled receptors (GPCRs) has been extensively studied as a trigger for lysosomal degradation and downregulation of signaling cascades (Critchley et al., 2018; Dores and Trejo, 2019). However, in specialized contexts, ubiquitylation can exert signal-promoting functions instead (Zhang et al., 2017). Non-signaling integral PM residents, including ion channels (Estadella et al., 2020) and amyloid precursor proteins (Gireud-Goss et al., 2020), are also subject to Ub-dependent lysosomal turnover. Moreover, ubiquitylation has also been shown to guide the delivery and maturation of proteolytic enzymes in acidic compartments (Gonzalez et al., 2018), as well as regulate transport of biosynthetic cargoes between the *trans*-Golgi network (TGN) and endosomes (De Angelis Rigotti et al., 2017; MacDonald et al., 2014). With these insights, our appreciation of Ub as the orchestrator of endolysosomal traffic and membrane homeostasis continues to evolve.

Regulation of the mammalian cell surface proteome is enforced by a conserved group of Ub ligases. Prominent among these are the RING E3s of the Casitas B-lineage lymphoma (Cbl) (Liyasova et al., 2015) and membrane-associated RING-CH (MARCH) (Lin et al., 2019) families, as well as the HECT E3s neuronal precursor cell-expressed developmentally downregulated 4 (Nedd4) (Sicari et al., 2022) and Itchy homolog (ITCH) (Aki et al., 2015; Moser and Oliver, 2019). Ub marks conjugated to cell surface molecules usually take the form of multiple monoubiquitins or K63-linked polyubiquitin chains; however, other linkage types such as K48 and K11 have also been reported (Haglund et al., 2003; Huang et al., 2006). Ub ligase activities governing turnover of the cell surface proteome are in turn countered by DUBs and can themselves be regulated by Ub and Ubls (Nelson et al., 2016; Polo, 2012; Wang et al., 2020). Collectively, these checks and balances ensure that physiological outcomes of Ub modification cascades are finetuned in accordance with fluctuating cellular demands. In the following section, we delve into the mechanisms employed by the cell to recognize Ub and Ubl conjugates on endosomal proteins and explore their consequences for the determination of cargo fate.

### Ub and SUMO as cargo fate determinants

Constitutive and ligand-induced internalization of cell surface molecules can occur via clathrin-mediated as well as clathrin-independent endocytic routes (Thottacherry et al., 2019). Much of what we know about the relationship between ubiquitylation and trafficking of endocytosed cargoes comes from studies on the epidermal growth factor receptor (EGFR) (Madshus and Stang, 2009) whose ligand-mediated activation leads to ubiquitylation of its cytoplasmic tails in a dose-dependent manner (Levkowitz et al., 1998; Sigismund et al., 2005). Whereas internalized EGFR molecules lacking Ub marks either quickly recycle back to the PM or progress gradually along the endosomal maturation route to accommodate steady state receptor turnover, robustly ubiquitylated receptors are actively committed to degradation (Holler and Dikic, 2004; Sigismund et al., 2013), as described below (Fig. 1A–C).

Recognition of ubiquitylated cargoes like EGFR is mediated by molecular machineries (Piper et al., 2014) in possession of Ub-binding domains (UBDs) and Ub-interacting motifs (UIMs) (Husnjak and Dikic, 2012). At the earliest stages of endocytosis, adaptors belonging to the EGFR pathway substrate 15 (EPS15) family interact with ubiquitylated receptors, directing their inclusion into nascent endosomes (Day et al., 2021) (Fig. 1A,B). Additionally, noncanonical modes of ubiquitylated cargo recognition at the PM are beginning to emerge (Li et al., 2017). Sorting of ubiquitylated cargoes takes place at the multifunctional SE compartment, which harbors both recycling and maturing membrane subdomains (Norris and Grant, 2020). Here, endosomal sorting complexes required for transport (ESCRTs) cluster ubiquitylated membrane proteins and execute their deposition into ILVs (Vietri et al., 2020) (Fig. 1C). The ESCRT-0 complex, consisting of the hepatocyte growth factor regulated substrate (HRS) and the signal transducing adaptor molecules 1 and 2 (STAM1 and STAM2) (Bache et al., 2003), mediates ubiquitylated cargo recognition (Raiborg et al., 2002; Roxrud et al., 2008) using its UIM (Shih et al., 2002) and VHS domains (Ren and Hurley, 2010). In line with the commonly observed signals for lysosomal degradation of transmembrane proteins, STAMs prefer K63-linked polyubiquitin chains (Lange et al., 2012). Subsequently, ESCRT-I, -II and -III act in succession to curve the limiting membrane around the cargoes selected by ESCRT-0 and perform scission of nascent ILVs into the endosomal lumen (Hurley, 2015; Tang et al., 2016). ESCRTs operating downstream of cargo selection are also capable of Ub binding (Shields et al., 2009), which ensures that sorting and ILV formation proceed as coupled processes. Importantly, the dynamic nature of endocytic progression requires ubiquitylation to be reversible (Clague et al., 2012). This activity is provided by DUBs that oppose Ub-dependent lysosomal degradation (Box 1) and modulate localization and function of endosomal proteins through mechanisms detailed in the upcoming section.

**Box 1. To DUB or not to DUB? That is the question!**

The reversible nature of Ub conjugation endows the processes it regulates with a dynamic character. Given the pervasiveness of Ub-mediated regulation in endosome biology and cargo traffic, it is not surprising that proteases responsible for Ub cleavage are critical for the integrity and physiology of endolysosomal systems across the eukaryotic kingdom (McCann et al., 2016). In yeast, the ubiquitin proteases Ubp2 and Ubp15 limit ubiquitylation and degradation of ARTs (Ho et al., 2017), and Ubp4 (also known as Doa4) recycles ubiquitin from endosomal cargoes prior to their inclusion into ILVs en route to the vacuole (Richter et al., 2007). In mammals, the latter function is likely performed by USP8 (Row et al., 2006), whereas the former is shared by multiple DUBs from different families. USP8 also features prominently in the context of cargo recycling, where it deubiquitylates various RTKs and thus diverts them away from lysosomal proteolysis (Berlin et al., 2010; Niendorf et al., 2007). Similarly, deubiquitylation by USP10 stimulates recycling of the cystic fibrosis transmembrane conductance regulator (CFTR) (Bomberger et al., 2009) and (together with USP7) the sodium-hydrogen exchanger 3 (NHE3; also known as SLC9A3) channel (Han and Yun, 2020), while USP9x and ubiquitin C-terminal hydrolase L1 (UCHL1) promote recycling of α5β1 integrin receptor complexes (Kharitidi et al., 2015) and MHC class I (Reinicke et al., 2019), respectively.

DUB-mediated stabilization of the cell surface proteome has direct implications for signal transduction, such as higher receptor availability and failure to degrade activated receptor complexes. Both of these effects can promote cancer, as exemplified by USP8, whose oncogenic properties are well established in Cushing's disease (Islam et al., 2021; Perez-Rivas and Reincke, 2016). However, targeting DUBs in cancer is complicated by the fact that they are involved in different cellular pathways. For instance, another cancer-associated DUB, USP15, deubiquitylates transforming growth factor-β type I receptor (TGFβRI) and thus augments Smad-mediated signaling during cancer cell metastasis (Eichhorn et al., 2012), while also attenuating insulin-like growth factor signaling (Fukushima et al., 2017). These examples underscore both the therapeutic potential of DUB interference in disease and the likely challenges associated with such pursuits.

As noted in the introductory remarks, not all intralumenal cargoes are destined for the lysosome, and some ILVs are expelled as EVs into extracellular space (Raposo and Stoorvogel, 2013). Selection of EV cargoes during MVB biogenesis relies heavily on PTMs (Atukorala and Mathivanan, 2021; Carnino et al., 2020), wherein conjugation of SUMO proteins (rather than Ub) appears to play a prominent role. For example, SUMOylation of α-synuclein aggregates enhances their excretion and pathogenic dissemination via exosomes in Parkinson's disease (Kunadt et al., 2015). Similarly, SUMOylation of RNA-binding protein hnRNPA1 facilitates inclusion of long noncoding RNAs into EVs (Chen et al., 2021) (Fig. 1D). Against the backdrop of Ub-mediated commitment of cell surface proteins for degradation, the emerging roles for SUMO in EV biogenesis illustrate that much remains to be learned about the stratification of endolysosomal cargo fates.

### Regulation of endocytic machineries by Ub and Ubls

The dynamic nature of membrane remodeling and cargo sorting requires the molecular machineries that perform these tasks to repeatedly associate with and disengage from their target membranes. For endosomal adaptor proteins, this cycling is afforded by an elegant regulatory loop, wherein their Ub recognition determinants switch between cargo binding (*in trans*) and auto-inhibition (*in cis*) (Hoeller et al., 2006). Specifically, monoubiquitylation of EPS15 leads to reversible inhibition of its UIM, rendering it temporarily unable to interact with ubiquitylated cargoes (Gschweitl et al., 2016) (Fig. 1B). Given that other UIM-containing endosomal adaptors, such as HRS and STAMs, are also subject to ubiquitylation, autoinhibition is likely to constitute a general mechanism for spatiotemporal control of endosomal machinery. Interestingly, the UIM of STAM overlaps with a SUMO-interacting motif (SIM) (Guzzo and Matunis, 2013), suggesting that recognition of mixed chains containing both ubiquitin and SUMO could further finetune ESCRT-0 function. Other sorting proteins are also subject to modifications with Ub and Ubls, as exemplified by the ESCRT-I core component TSG101 (also known as VPS23). An important regulator of endocytic and exocytic traffic in plants and mammals, TSG101 is both ubiquitylated and modified by another ubiquitin-like molecule, namely ISG15, leading either to proteasomal degradation (Xia et al., 2020) or aggregation and disposal in the autolysosome (Villarroya-Beltri et al., 2016), respectively. Both outcomes transiently mimic ESCRT-I loss-of-function, resulting in impairment of MVBs biogenesis and EV secretion. Collectively, the above examples illustrate various ways in which functions of sorting complexes are regulated by the interplay between Ub and Ubl recognition and conjugation, which can be further finetuned through interferon release during inflammation.

Because addition of Ub and Ubls to cargoes and sorting proteins cannot on its own support the dynamic interplay between them, timely action of DUBs is required to complete the cycle of Ub-dependent trafficking control. Thus far, only a few DUBs have been described to directly target endosomal adaptors, thus regulating their stability and function. These include USP9x, which deubiquitylates EPS15 to relieve its autoinhibition and thereby promotes trafficking and downregulation of ubiquitylated receptors (Savio et al., 2016) (Fig. 1B). At the SE, two other DUBs USP8 and AMSH (also known as STAMBP) are recruited to the Src homology 3 (SH3) domains of STAM1 and STAM2 (Kato et al., 2000; McCullough et al., 2004) (Fig. 1B), enabling them to deubiquitylate the ESCRT-0 complex and consequently ensure the continuity of sorting function at the SE (Berlin et al., 2010; Sierra et al., 2010). USP8, also known as UBPY, is an essential protein in mammals that is required for embryonic stem cell renewal (Gu et al., 2019), growth factor receptor maintenance (Niendorf et al., 2007) and T cell development and homeostasis (Dufner et al., 2015). In T cells, USP8 foregoes binding to STAM proteins for the higher affinity SH3 domain of growth factor receptor-bound protein 2-related adaptor downstream of Shc (GADS; also known as GRAP2), demonstrating how varying the strength of modular interactions achieves functional diversification in specialized cell types. Additionally, USP8, as well as AMSH, also interact with ESCRT-III adaptors, including the accessory protein tyrosine phosphatase non-receptor type 23 (PTPN23) and charged MVB proteins (CHMPs) (Adoro et al., 2017; Ali et al., 2013; Kyuuma et al., 2007) (Fig. 1C), which enable these DUBs to participate in late endosomal sorting steps. In contrast to the linkage-promiscuous USP8 (Durcan and Fon, 2015; Row et al., 2006; Zheng et al., 2018), AMSH exhibits specificity for K63 polyubiquitin chains (Davies et al., 2011; Sato et al., 2008). This preference is shared with STAM, whose binding to AMSH promotes its DUB activity and dictates the position of Ub cleavage (Baiady et al., 2016; Hologne et al., 2016). Together, the ESCRT-associated DUBs are ideally positioned to globally survey the molecular decisions taking place at the SE and ensure accurate and efficient cargo flow.

**Fig. 2.**
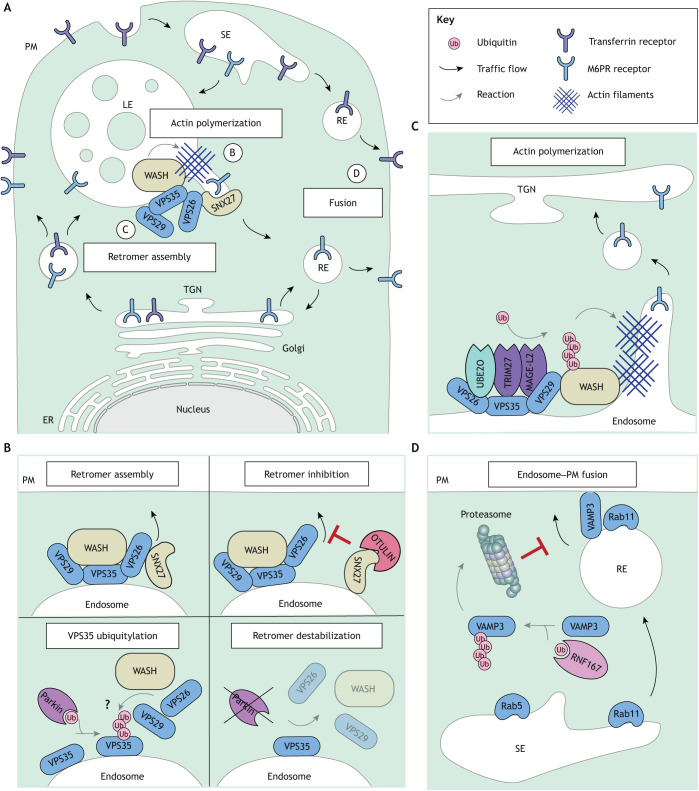
**Ubiquitylation regulates retromer-mediated recycling.** (A) Membrane recycling from maturing endosomes towards the plasma membrane (PM) and the trans-Golgi network (TGN) is carried out by the retromer complex comprising the cargo-selective subunit VPS35 and its partners VPS29 and VPS26. The retromer associates with the WASH complex for actin filament assembly and membrane-deforming sorting nexins (SNX) proteins. Processes labeled B–D are shown in more detail in the other panels. (B) Retromer assembly and stability are regulated by ubiquitylation. The E3 ligase Parkin ubiquitylates VPS35 and stabilizes other retromer components, however, whether ubiquitylation of VPS35 promotes retromer assembly is unclear. Association of the DUB OTULIN with SNX27 antagonizes its binding to VPS26 and thus inhibits retromer-mediated recycling to the PM. (C) Ubiquitylation of the WASH complex, mediated by the retromer-associated E3 ligase MAGE-L2 in conjunction with its substrate adaptor TRIM27 and conjugating E2 enzyme UBE2O, activates localized actin polymerization and promotes recycling from endosomes to the PM. (D) Polyubiquitylation of the SNARE VAMP3 by the E3 ligase RNF167 enhances proteasomal destruction of VAMP3 and inhibits fusion of TGN-derived recycling compartments with the PM.

Besides ESCRTs, other endolysosomal machineries are also subject to regulation by Ub and Ubls. One such example is the retromer complex responsible for membrane recycling from maturing endosomes (Chen et al., 2019) (Fig. 2A). Retromer comprises the core subunits VPS35, VPS26 and VPS29, which work together with membrane deforming sorting nexins (SNXs) (Hanley and Cooper, 2020) and the Wiskott–Aldrich syndrome protein and SCAR homolog (WASH) actin-polymerizing complex (Simonetti and Cullen, 2019) to identify and extract membranes carrying recycling cargoes such as the mannose-6-phosphate receptor (M6PR) to the Golgi and TGN, or the β2-adrenergic receptor to the cell surface (Seaman, 2012). Retromer complex assembly on endosomal membranes is modulated by the ovarian tumor DUB with linear linkage specificity (OTULIN) whose recruitment by SNX27 antagonizes the interaction between SNX27 and VPS26 and thus inhibits cargo loading (Stangl et al., 2019) (Fig. 2B). Furthermore, the cargo-selective retromer component VPS35 interacts with several Ub-modifying enzymes, including the RING adaptor melanoma-associated antigen gene L2 (MAGE-L2), which together with the conjugating enzyme UBE2O and the tripartite motif protein 27 (TRIM27) mediates K63-linked polyubiquitylation of WASH (Hao et al., 2013). This stimulates WASH to produce localized actin filaments at sites of tubule formation and facilitates cargo retrieval from endosomes (Fig. 2C). VPS35 is itself ubiquitylated by another Ub ligase (Fig. 2B), the RING/HECT hybrid Parkin (Williams et al., 2018). Both Parkin and VPS35 are implicated in the pathogenesis of Parkinson's disease (Panicker et al., 2021), and, although the mechanistic consequences of their interplay remain unclear (Williams et al., 2017), Parkin appears to promote retromer complex integrity, as brains of Parkin-knockout mice show marked depletion of retromer-associated WASH proteins (Williams et al., 2018). Interestingly, Parkin also cooperates with the endosomal Ub adaptor Toll-interacting protein (TOLLIP) (Katoh et al., 2004) in trafficking of stress-induced mitochondrial-derived vesicles for lysosomal clearance in a retromer-dependent manner (Ryan et al., 2020). These findings reinforce the notion that Ub-mediated interactions are broadly applicable across many membrane pathways feeding into the endolysosomal system, all of which serve important roles in the regulation of cellular homeostasis.

Another aspect of endosome dynamics regulated by Ub is membrane fusion (Gautreau et al., 2014), which is orchestrated by soluble N-ethylmaleimide-sensitive factor attachment protein receptors (SNAREs) (Yoon and Munson, 2018). Examples of SNARE–Ub interplay include inhibition of recycling from early endosomes through ubiquitylation of vesicle-associated membrane protein 3 (VAMP3) by the RING-finger protein 167 (RNF167) (Yamazaki et al., 2013) and atypical Ub recognition by syntaxin 3 (Giovannone et al., 2020) (Fig. 2D). To accomplish fusion, SNAREs collaborate with membrane tethering complexes class C core vacuole/endosome tethering (CORVET) and homotypic fusion and vacuole protein sorting (HOPS) (Balderhaar and Ungermann, 2013), both of which contain VPS11 and VPS18 subunits with RING domains capable of E3 Ub ligase activity (Segala et al., 2019; Yogosawa et al., 2005). *In vitro* reconstitution of the HOPS complex suggests that these RING domains are embedded within the structure and might be unavailable for ligase function (Shvarev et al., 2022 preprint). However, several CORVET and HOPS subunits have been described to function independently (van der Beek et al., 2019), allowing for the possibility of Ub ligation by VPS11 and/or VPS18 in other cellular contexts.

Collectively, these studies illustrate how selective engagement of Ub and Ubl modifying enzymes by executors of endosomal traffic controls fundamental processes in vesicle biology, namely, fusion, fission and cargo flow. Successful completion of these tasks depends in turn on appropriate designation, maintenance and exchange of vesicular membrane identity. How this aspect of endolysosomal biology is modulated by Ub and Ubls is discussed in the following section.

## Ub and Ubls as influencers of membrane dynamics

### Smooth transitions – ubiquitylation and SUMOylation regulate endolysosomal membrane identity

**Fig. 3.**
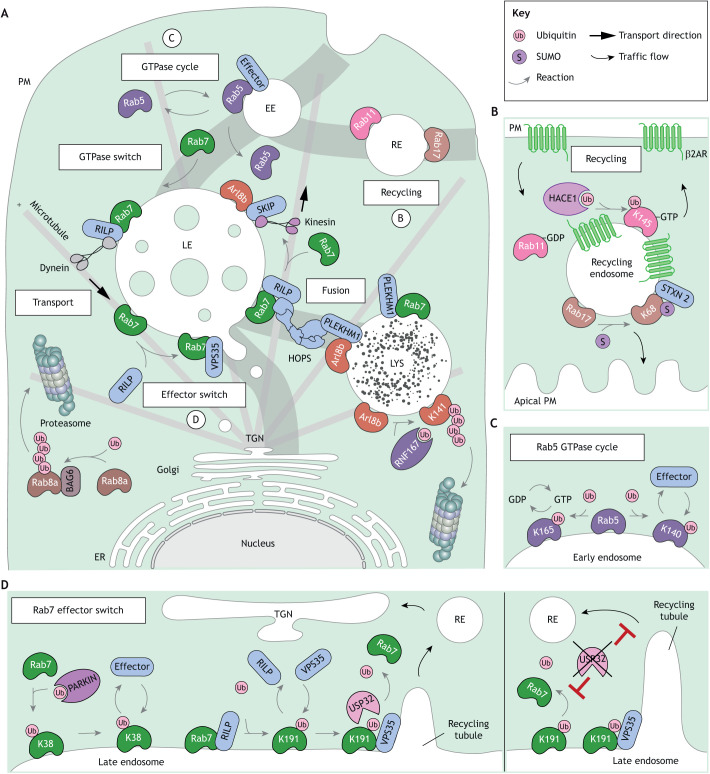
**Ub and Ubls in endosomal membrane identity transitions.** (A) Regulated switches between small GTPases determine endosome identity, transport and fusion through selective effector recruitment. During maturation, late endosome (LE)-associated Rab7 displaces early endosome (EE)-associated Rab5. Rab7 engages the effector RILP and the dynein motor to direct transport of late endosomes (LEs) to the perinuclear region. Here, HOPS-mediated tethering and fusion with lysosomes carrying Rab7 and/or Arl8b in complex with the effector PLEKHM1 takes place. On the other hand, displacement of Rab7 by Arl8b in complex with effector SKIP allows for kinesin motor-dependent transport to the cell periphery. Polyubiquitylation of Arl8b by E3 ligase RNF167 leads to its degradation by the proteasome. Other small GTPases including Rab8a are also turned over by the proteasome in a manner dependent on the cytosolic chaperone BAG6. Processes labeled B–D are shown in more detail in the other panels. (B) Ub and Ubl conjugation regulates Rab-mediated recycling. Endocytosed β2-adregergic receptor recruits the E3 ligase HACE1 to ubiquitylate and activate Rab11 for recycling to the plasma membrane (PM). Also, SUMOylation of Rab17 promotes SNARE syntaxin 2-mediated recycling to the apical surface. (C) Key consequences of GTPase ubiquitylation include altered GTP hydrolysis rate and effector interactions. This is illustrated by Rab5 whose monoubiquitylation on different lysine residues affects distinct aspects of its functional cycle. (D) Left, reversible ubiquitylation of Rab7 by the E3 ligase Parkin and DUB USP32 regulates effector preference. Monoubiquitylation of Rab7 disfavors interaction with RILP and promotes association with the retromer complex. Right, loss of USP32 inhibits recycling tubule resolution and blocks endosomes-to-TGN traffic. Acceptor lysine residues are indicated within some shapes.

Decisive commitment of membranes and cargoes to their appropriate trafficking trajectories requires originating membranes to display different characteristics from those found at the destination site (Weeratunga et al., 2020). Intrinsic membrane identity is defined by distinct phospholipid signatures (Schink et al., 2016). In the endolysosomal system, established early and sorting compartments carry phosphatidylinositol 3-phosphate (PI3P), which is converted into phosphatidylinositol 3,5-bisphosphate (PI3,5P_2_) during maturation (Cullen and Carlton, 2012). Different PI species in turn drive membrane occupancy by small GTPases (Wandinger-Ness and Zerial, 2014) of the Rab (Homma et al., 2021), Arf and Arl (Sztul et al., 2019) families, whose ability to switch between active (GTP-bound) and inactive (GDP-bound) states by way of GTP hydrolysis underlies the dynamic character of membrane traffic (Pfeffer, 2017). Once activated, endosomal GTPases exert their functions through recruitment of specialized effectors that couple transport, fusion and fission machineries to their membranes of choice (Langemeyer et al., 2018). To accommodate smooth progression along the endolysosomal track, endosomes exchange their GTPases through regulated hand-over mechanisms. These include Rab5-to-Rab7 conversion on maturing endosomes (Langemeyer et al., 2020; Poteryaev et al., 2010), followed by Rab7-to-Arl8b exchange on degradative compartments (Jongsma et al., 2020; Marwaha et al., 2017) (Fig. 3A).

The activity cycle of small GTPases is extensively influenced by PTMs (Shinde and Maddika, 2018), and the relevance of Ub and Ubls is rapidly emerging in this context. The first ubiquitylation on an endosomal GTPase was described for Rab11 (which has Rab11a and Rab11b isoforms), responsible for recycling from EEs, demonstrating that monoubiquitylation of Rab11 on K145 promotes recycling of β_2_-adrenergic receptor to the PM (Lachance et al., 2014) (Fig. 3B). Ubiquitylation of Rab11 effectors has also been implicated in cargo recycling (Sakai et al., 2019), underscoring the varied ways in which this PTM can influence the behavior and function of protein complexes on endosomes. Importantly, conjugation of Ub and Ubls to distinct lysine residues on the same GTPase can lead to different outcomes (Jung et al., 2022). For instance, monoubiquitylation of Rab5 (which has Rab5a, Rab5b and Rab5c isoforms) on K140 interferes with its binding to effectors Rabaptin5 (also known as RABEP1) and early endosome antigen 1 (EEA1), whereas the same modification on K165 affects the intrinsic GDP–GTP conversion cycle (Shin et al., 2017) (Fig. 3C). At later maturation stages, monoubiquitylation of Rab7 (which has Rab7a and Rab7b isoforms) on K38 by the E3 ligase Parkin influences its membrane association and effector interactions (Song et al., 2016). Additionally, monoubiquitylation of Rab7 on K191 (by an unknown ligase) destabilizes binding of the retrograde transport effector RILP responsible for dynein motor recruitment and promotes interaction with the retromer complex instead (Sapmaz et al., 2019) (Fig. 3D). Interestingly, both of the aforementioned Rab7 functions benefit from the deubiquitinating activity of USP32 (Sapmaz et al., 2019), suggesting that reversible ubiquitylation tunes the switching of Rab7 between its different effects in the endocytic pathway. Several endosomal GTPases are also subject to SUMOylation, including Rab7 (Mohapatra et al., 2019), Rab17 (Striz and Tuma, 2016) and Arl13b (Li et al., 2012). However, whether SUMOylation constitutes a general mode of small GTPase control remains to be established.

Besides modulating membrane association, effector binding and GTP hydrolysis, ubiquitylation can also stimulate GTPase turnover by the proteasome, as shown for Arl8b (Deshar et al., 2016) (Fig. 3A), Rab35 (Villarroel-Campos et al., 2016), Rab27a (Song et al., 2019) and Arl4C (Han et al., 2020). Furthermore, it has been speculated that some small GTPases interact with the BAG6 chaperon/holdase cytosolic quality control complex when in the GDP-bound state (Takahashi et al., 2019), and that this might constitute a general mechanism for clearance of inactive Rabs. Taken together, the above examples illustrate how Ub and Ubls offer timely controls over small GTPase abundance and function. The notion that such reversible modifications are crucial in timing dynamic membrane behaviors is further explored below where we describe the emerging roles of Ub and Ubls in formation and dissolution of specialized organellar interaction hubs termed membrane contact sites (MCSs).

### Touch and go – ubiquitylation at MCSs

**Fig. 4.**
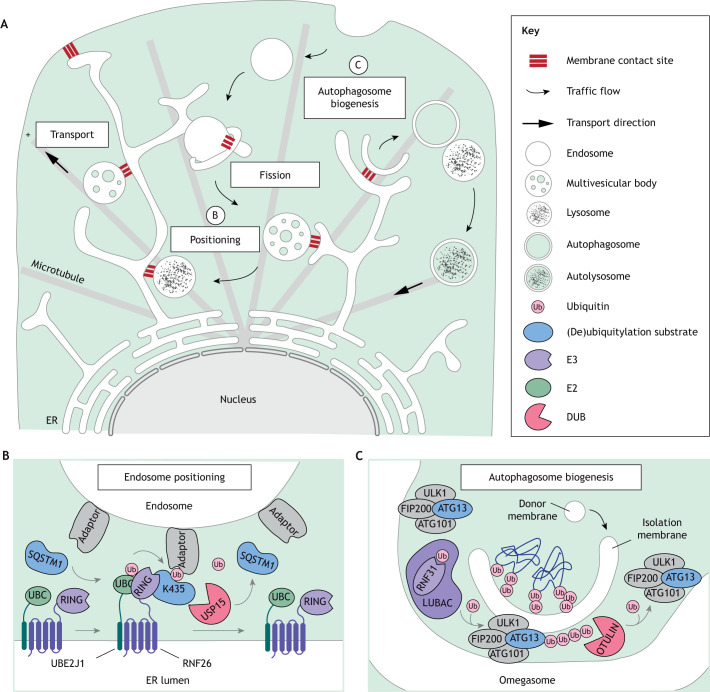
**Reversible ubiquitylation at membrane contact sites.** (A) ER membrane contact sites (MCSs) regulate endocytic traffic by directing endosome transport, specifying location and timing of endosome fission, and positioning endosomes (and lysosomes) in the perinuclear space. ER MCSs are also involved in autophagosome biogenesis. Processes labeled B and C are shown in more detail in the other panels. (B) Ub-dependent reversible ER-endosome MCS formation. The membrane-embedded UBE2J1–RNF26 E2–E3 pair ubiquitylates SQSTM1 on K435 to position endosomes at the perinuclear ER. Deubiquitylation by USP15 in turn dissolves this MCS, releasing endosomes for fast transport. (C) Regulation of early steps in autophagosome biogenesis by the ER. The ULK1 complex localizes at omegasome regions of the ER membrane, where it stimulates formation of the isolation membrane (IM). Timing of IM elongation is controlled by the E3 ligase RNF31 of the LUBAC complex, which mediates linear polyubiquitylation of ATG13. Once the autophagosomal double-membrane is sealed, deubiquitylation of ATG13 by OTULIN promotes autophagosome maturation.

An abundance of physical interactions takes place between intracellular membranes in the form of MCSs (Prinz et al., 2020), which provide platforms for organellar communication and material exchange (Scorrano et al., 2019). The majority of MCSs involve the endoplasmic reticulum (ER) – an organelle whose dynamic membrane network is ideally suited to coordinate diverse cellular pathways and balance homeostasis (Nixon-Abell et al., 2016; Spang, 2018). The ER interacts extensively with endosomes, lysosomes and autophagosomes, guiding their motility, fusion and fission through various types of MCSs (Dong et al., 2016; Hoyer et al., 2018; Raiborg et al., 2015; Rocha et al., 2009; Wijdeven et al., 2016) (Fig. 4A). Thus far, only one example of Ub-dependent MCS formation between endocytic compartments and the ER has been reported (Cremer et al., 2021; Jongsma et al., 2016). Here, the RING E3 ligase RNF26, embedded within the perinuclear ER subdomain anchored by vimentin intermediate filaments (Cremer et al., 2022 preprint), cooperates with the conjugating enzyme UBE2J1 to modify the cytosolic Ub adaptor SQSTM1 (Fig. 4B). The resulting Ub-rich complex engages UBDs of endolysosomal adaptors, such as TOLLIP and EPS15, to position their cognate vesicles in the perinuclear cytoplasm. This action can subsequently be reversed by the DUB USP15, releasing vesicles for fast transport into the cell periphery. Together, these enzymes help maintain the organization of the entire endosomal repertoire (including vesicles of the TGN) in space and time, and, in so doing, facilitate efficient endolysosomal maturation and downregulation of extracellular signals (Cremer et al., 2021; Jongsma et al., 2016). Interestingly, prior to their deposition into ILVs, activated receptors, such as EGFR, undergo dephosphorylation at ER–endosome MCSs, mediated by the ER-associated phosphatase PTP1B (also known as PTPN1) (Eden et al., 2010). However, whether RNF26 and PTP1B collaborate with respect to EGFR downregulation remains unknown. Furthermore, given that the ER membrane harbors many ubiquitin ligases (Fenech et al., 2020), it is possible that additional mechanisms involving ubiquitin conjugation operate at MCSs between the ER the various members of the endolysosomal system.

Besides guiding endolysosomal maturation and dynamics, ER MCSs are also instrumental during early steps of autophagosome biogenesis (Kohler et al., 2020). In mammalian cells, autophagosomes are thought to originate at omega-shaped regions of the ER in response to the Unc-51-like autophagy activating kinase 1 (ULK1) (Feng et al., 2014; Nascimbeni et al., 2017). These early steps are coordinated through short-lived MCSs between the ER and the growing isolation membrane (IM), which are tethered by integral ER proteins VAPA and VAPB and IM-associated WD repeat domain phosphoinositide-interacting protein 2 (WIPI2), as well as the autophagy-related protein 2 (ATG2)–WIPI4 (WIPI4 is also known as WDR45) tethering complex (Graef, 2018; Zhao et al., 2018). To allow sufficient time for IM expansion, the linear ubiquitin assembly complex (LUBAC) modifies and stabilizes ATG13, a component of the ULK complex (Fig. 4C). Once the double-membrane of the phagophore seals around the designated (ubiquitylated) cargoes, deubiquitylation of ATG13 by OTULIN untethers the MCS, allowing the autophagosome to mature. Here again, reversible ubiquitylation provides temporal control of dynamic membrane remodeling events through short-lived interorganellar contacts. Notably, VAPA- and VAPB-mediated ER MCSs also dictate autophagosome transport and fusion with the endolysosomal system (Wijdeven et al., 2016), yet whether reversible modifications with Ub and Ubls affect these aspects is unknown. In the same vein, ER MCSs have been implicated in the maturation of phagosomes formed around intracellular bacterial invaders (Derre, 2017). Involvement of Ub and Ubls in this context (unexplored to date) is likely, given that pathogenic bacteria are expert usurpers of host Ub and Ubl conjugation cascades, as described in the section below.

## Bacteria exploit Ub and Ubls to reprogram the endolysosomal system of the host

**Fig. 5.**
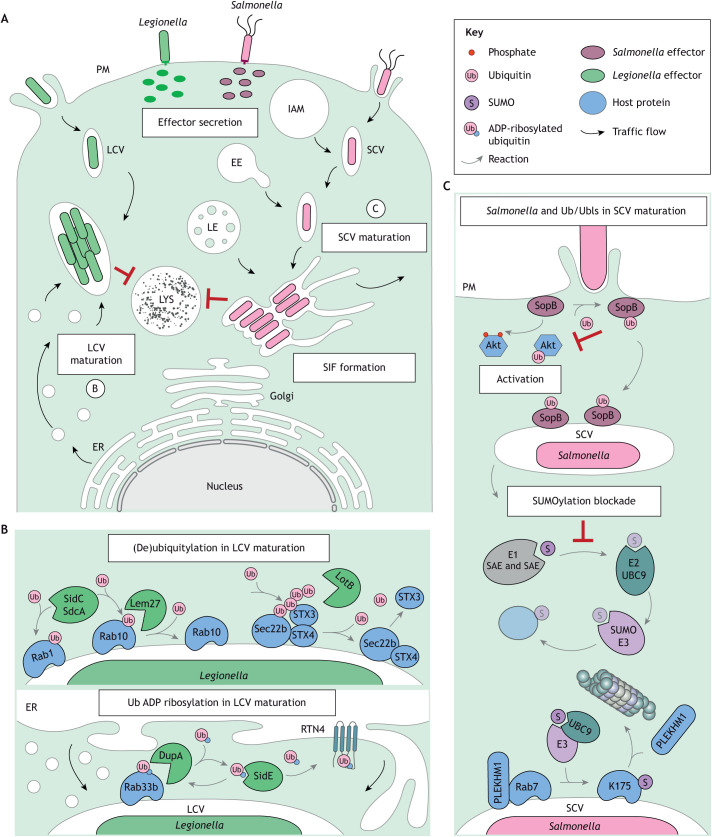
**Hijacking of Ub and Ubls by intracellular bacteria to reprogram the endolysosomal system of the host.** (A) Intracellular bacteria exploit reversible Ub and Ubl conjugation to reprogram the host endolysosomal system in ways benefitting their infection cycle. Both *Legionella* and *Salmonella* establish vacuolar compartments that mature into replication permissive niches while avoiding lysosomal degradation. IAM, infection associated macropinosome; EE, early endosome; LE, late endosome; LYS, lysosome. Processes labeled B and C are shown in more detail in the other panels. (B) Following bacterial entry, *Legionella* exploits the host ubiquitylation machinery, as well as secreting its own unique repertoire of enzymes that enable the *Legionella*-containing vacuole (LCV) membrane to acquire ER-like characteristics and evade the endolysosomal system. This strategy includes ubiquitylation of GTPases Rab1 and Rab10, temporal regulation of membrane expansion by reversible ubiquitylation of the SNARE Sec22b, and promotion of LCV maturation through noncanonical modifications of host factors with ADP-ribosylated Ub. (C) Ubiquitylation of the *Salmonella* effector SopB directs it to the *Salmonella*-containing vacuole (SCV), where it regulates membrane identity dynamics at the SCV. Conversely, the non-ubiquitylated form of SopB localizes predominantly to the PM and instigates activation of Akt signaling. *Salmonella* infection also induces a global SUMOylation blockade in the host cell through downregulation of the SUMO-conjugating enzyme UBC9. This stabilizes the key endolysosomal Rab7 that promotes maturation of the SCV. Acceptor lysine residues are indicated within some shapes.

Many intracellular pathogens have evolved sophisticated strategies of subverting host cell processes to benefit their infection programs. Notable among these tactics is manipulation of Ub and Ubl signaling to reprogram host cell organization in service of pathogen uptake and dissemination (Ashida et al., 2014; Zhou and Zhu, 2015). From the moment of infection, bacteria, as well as viruses, exploit the host Ub system to enhance their entry. For instance, *Listeria*, the bacterium causing listeriosis, stimulates ubiquitylation of host cell surface receptors E-cadherin and Met, which recruit the machinery for clathrin-mediated endocytosis and promote actin cytoskeleton rearrangements necessary for bacterial internalization (Pizarro-Cerda et al., 2012). A similar strategy is exploited by the dengue virus, whose entry into T cells necessitates ubiquitylation of its receptor immunoglobulin mucin 1 (TIM-1; also known as HAVCR1) (Dejarnac et al., 2018), or the Zika virus, whose envelope protein ubiquitylation increases binding to host receptors (Giraldo et al., 2020). Once inside the host cell, some pathogens strategically remain within a membrane-bound compartment (Fig. 5A), which matures into a supportive replicative niche through active remodeling of host membranes, selective harvesting of nutrients and avoidance of degradation. Below we discuss *Legionella* and *Salmonella* as examples of bacterial species that have mastered the art of hijacking host membrane traffic and Ub and Ubl conjugation pathways to build and sustain their replicative niche.

### *Legionella* – a master of Ub manipulation

Notably, *Legionella pneumophila*, the causative agent of Legionnaire's lung disease, secretes an arsenal of Ub ligases and DUBs, some of which manipulate vesicular traffic. For instance, the *Legionella* Ub ligases SidC and SdcA alter localization and activity of host Rab1 and Rab10 proteins (Jeng et al., 2019) (Fig. 5B). Ubiquitylation of Rab10 is further counteracted by the DUB effector Lem27, and this interplay guides the maturation of the *Legionella*-containing vacuole (LCV) in space and time (Liu et al., 2020). *Legionella* also promotes sequential addition and removal of Ub from the host SNARE Sec22b (Kitao et al., 2020). Collectively, these efforts enable the LCV to acquire ER-like characteristics and evade the endolysosomal system.

Not only does *Legionella* express conventional ligase and DUB effectors that cooperate with the Ub machinery of the host (Qiu and Luo, 2017), it also deploys a unique set of unconventional enzymes that utilize Ub without the need for host E1 or E2 activities (Bhogaraju and Dikic, 2016). Specifically, effectors of the SidE family conduct ADP-ribosylation of R42 on the host Ub, rendering it competent for direct attachment to serine residues of protein substrates (Qiu et al., 2016). This noncanonical Ub PTM impairs conventional ubiquitylation (Bhogaraju et al., 2016) and promotes maturation of the LCV by targeting host traffic regulators Rab33b and the ER reticulon protein Rtn4 (Kawabata et al., 2021; Kotewicz et al., 2017) (Fig. 5B). To complete this PTM cycle, *Legionella* also encodes phosphodiesterase effectors DupA and DupB, which reverse SidE-mediated ubiquitylation, ensuring the identity of the LCV membrane remains dynamic over time (Qiu et al., 2017). Whether other organisms can also reversibly modify proteins with ubiquitin via phosphoribosylation is unclear.

### *Salmonella* rewires endolysosomal traffic via SUMO and Ub

Another intracellular bacterium, *Salmonella* Typhimurium, which causes gastroenteritis, takes a different global approach. *Salmonella* induces a SUMOylation blockade in the host cell by downregulating UBC9 (Verma et al., 2015). One key consequence of this blockade is the stabilization of Rab7 and enhanced binding to the fusion effector PLEKHM1, which is needed for the *Salmonella*-containing vacuole (SCV) to mature into a permissive replicative niche (Mohapatra et al., 2019) (Fig. 5C). Beyond Rab7, these findings raise new questions regarding the potential for broader implications of SUMOylation on the host–pathogen interaction landscape. In particular, considering that the SCV membrane undergoes several identity transitions prior to arriving at the Rab7-positive stage (Smith et al., 2007), the findings described above might represent merely the proverbial tip of the iceberg when it comes to Ub and Ubl-guided GTPase control of the SCV.

Localization and activity of bacterial effector proteins can also be regulated by Ub and Ubls. This is exemplified by the *Salmonella* effector SopB, which controls membrane identity of the PM, and subsequently the SCV, through phosphorylation of host phosphoinositides (Hernandez et al., 2004; Walpole et al., 2022). These sequential and locally restricted functions of SopB in promoting bacterial uptake and maturation of the SCV, respectively, are coordinated by a shift in SopB ubiquitylation (Fig. 5C). Specifically, SopB-dependent actin remodeling necessary for bacterial internalization is intensified and sustained by a SopB mutant lacking Ub acceptor lysine residues (Knodler et al., 2009; Patel et al., 2009). Conversely, ubiquitylation of SopB inhibits its early activity at the PM, prolonging the retention of this effector on the SCV instead (Knodler et al., 2009; Patel et al., 2009). In this way, *Salmonella* exploits the Ub and Ubl machinery to steer host and bacterial protein activities in space and time, tuning the endolysosomal landscape to optimally serve its own needs.

### Ub and Ubls tune the host response

A key defense against intracellular bacterial invaders is provided by the autophagy-like pathway termed xenophagy, meaning ‘foreign-eating’ (Knodler and Celli, 2011). Cytosolic bacteria, damaged bacteria-containing vacuoles and membrane remnants resulting from vacuolar disruption can all be targeted for ubiquitylation by the LUBAC complex, marking them for autophagic clearance (Tripathi-Giesgen et al., 2021). To circumvent this fatality, the cytosolic bacterium *Shigella flexneri* secretes IpaH family E3 ligases that ubiquitylate and degrade LUBAC, thereby neutralizing the very machinery necessary for bacterial demise (de Jong et al., 2016; Liu et al., 2022). *Legionella*, on the other hand, deploys a DUB effector RavD to specifically hydrolyze linear Ub chains on the LCV (Wan et al., 2019). Using a similar strategy, *Salmonella* exploits a DUB effector SseL to remove Ub chains decorating cytosolic aggregates formed during infection, subduing autophagic flux and promoting bacterial replication (Mesquita et al., 2012). *Salmonella* also repurposes the host autophagy machinery to repair its vacuolar membrane (Kreibich et al., 2015). From the host perspective, macrophages activated by interferon γ unleash a Ub-dependent pathogen defense orchestrated by the E3 ligase RNF115 to slow maturation of the phagosome compartment and reduce *Salmonella* replication (Bilkei-Gorzo et al., 2022). Another interferon γ-induced E3 ligase, namely RNF213, ubiquitylates *Chlamydia* inclusion bodies for autolysosomal clearance, and this function is inhibited by the bacterial shielding protein GarD (Walsh et al., 2022). Collectively, these examples reveal how reversible ubiquitylation of intracellular bacteria and their phagosomal niche can be exploited by pathogens and host cells alike attempting to gain the upper hand in the conflict between them.

## Conclusions and perspectives

The sheer complexity and dynamic nature of intracellular traffic necessitates subtle but decisive controls over membrane identity and cargo fate. As our appreciation for various ways in which Ub and Ubls endow the endolysosomal system with spatial and temporal constraints evolves, many exciting questions come to the forefront. For instance, whether and how the interplay between intrinsic membrane properties (i.e. the phospholipid repertoire) and secondary factors, such as small GTPases and their effectors, is regulated by Ub and Ubls is unclear. Related to this, a comprehensive view of Ub and Ubls as modulators of intracellular bacterial niches, which are strongly reliant on membrane identity control, is presently lacking. With antibiotic resistance on the rise worldwide, identification of novel druggable targets from the side of the host as well as the pathogen may offer new routes for treatment of bacterial infections (Stevenin and Neefjes, 2022).

Another glaring unknown in the field is the molecular nature of internal membrane equilibrium within endosomes. Given the importance of Ub and Ubl dynamics during ILV formation, it is reasonable to hypothesize that reversal of this process through retrofusion might also be modulated by these PTMs. Moreover, because MVB dynamics have direct implications for EV biogenesis and intercellular communication in cancer metastasis and neurodegeneration, enquiry into Ub and Ubl (de)conjugating enzymes regulating endosomes dynamics has the potential to illuminate and eventually modulate these pathological processes. Meanwhile, rapid developments in proteomic approaches (Grandi and Bantscheff, 2019; Kliza and Husnjak, 2020), genome editing (Pickar-Oliver and Gersbach, 2019) and spatiotemporally resolved imaging techniques (Guo et al., 2018) are making it possible to identify and resolve dynamic cellular behaviors with greater ease and efficiency than ever before. Obtaining such insights at the crossroads of endocytosis and the Ub system constitutes an exciting challenge of the coming decade and holds promise for the development of new therapies.
